# Synbiotic Combination of Djulis (*Chenopodium formosanum*) and *Lactobacillus acidophilus* Inhibits Colon Carcinogenesis in Rats

**DOI:** 10.3390/nu12010103

**Published:** 2019-12-30

**Authors:** Chih-Wei Lee, Hong-Jhang Chen, Yu-Hua Chien, Shih-Min Hsia, Jiann-Hwa Chen, Chun-Kuang Shih

**Affiliations:** 1 School of Nutrition and Health Sciences, College of Nutrition, Taipei Medical University, Taipei 11031, Taiwan; leechihwei1012@gmail.com (C.-W.L.); betty3482@gmail.com (Y.-H.C.); bryanhsia@tmu.edu.tw (S.-M.H.); 2 Institute of Food Science and Technology, National Taiwan University, Taipei 10617, Taiwan; fsthjchen@ntu.edu.tw; 3 Graduate Institute of Metabolism and Obesity Sciences, College of Nutrition, Taipei Medical University, Taipei 11031, Taiwan; 4 School of Food Safety, College of Nutrition, Taipei Medical University, Taipei 11031, Taiwan; 5 Nutrition Research Center, Taipei Medical University Hospital, Taipei 11031, Taiwan; 6 Division of Gastroenterology and Hepatology, Department of Internal Medicine, Taipei Tzu Chi Hospital, New Taipei City 23142, Taiwan; cjhki.tiyi@msa.hinet.net; 7 Master Program in Food Safety, College of Nutrition, Taipei Medical University, Taipei 11031, Taiwan

**Keywords:** djulis, *Lactobacillus acidophilus*, synbiotics, colon cancer, apoptosis

## Abstract

Djulis is a functional grain containing prebiotic dietary fiber, which has an anti-cancer potential. This study examined the preventive effect of djulis alone or in combination with *Lactobacillus acidophilus* on colon carcinogenesis induced by 1,2-dimethylhydrazine (DMH) and dextran sulfate sodium (DSS). Rats were divided into five groups and fed B (AIN-93G, blank), C (AIN-93G, control), D (10% djulis), DLA (10% djulis plus 5 × 10^6^ cfu *L. acidophilus*/g), and DHA (10% djulis plus 5 × 10^7^ cfu *L. acidophilus*/g) diets, respectively. All rats except for those in group B received three doses of DMH (40 mg/kg) by intraperitoneal injection and 3% DSS in drinking water. After 10 weeks of feeding, the colon was analyzed for precancerous lesions and biomarkers. DMH and DSS treatment induced aberrant crypt foci (ACF), especially in the distal colon. D, DLA, and DHA significantly reduced the numbers of total ACF, sialomucin-producing ACF (SIM-ACF), and mucin-depleted foci (MDF) in the distal colon compared to C. Additionally, DLA and DHA further downregulated the expressions of proliferating cell nuclear antigen (PCNA) and cyclooxygenase-2 (COX-2) and regulated apoptosis-related proteins. These results suggest that synbiotic combination of djulis and *L. acidophilus* shows the best inhibitory effect on colon carcinogenesis via regulation of proliferative, inflammatory, and apoptotic pathways.

## 1. Introduction

Colorectal cancer (CRC) is the third most commonly diagnosed cancer worldwide [1]. Diet and lifestyle are closely associated with CRC. Previous studies pointed out that dietary pattern could significantly affect the risk of cancer [2]. Western diet, a diet high in fat and low in dietary fiber, increases the risk of CRC [3].

The development of CRC is a multiple and long-term process, including initiation, promotion, progression, and invasion. Inflammation and oxidative stress may initiate and promote CRC development. Cyclooxygenase-2 (COX-2) mediates inflammation and plays an important role in CRC. Increased COX-2 expression has been detected in both colorectal tumor-bearing animals [4] and CRC patients [5].

Overexpression of COX-2 can inhibit apoptosis, including both intrinsic and extrinsic pathways [6]. In CRC cells, reduction of B-cell lymphoma 2 (Bcl-2) protein expression and enhancement of Bcl-2-associated X (Bax) protein expression significantly inhibited cancer cell proliferation [7]. In addition, upregulation of caspase-3, caspase-8, and caspase-9 could promote apoptosis in HCT-116 cells [8]. These studies suggest that the increased expression of pro-apoptotic proteins and the decreased expression of anti-apoptotic proteins play a key role in the control of CRC.

Aberrant crypt foci (ACF), a precancerous lesion in the colon, have been generally accepted for use as a biomarker to investigate the effects of dietary factors on colon carcinogenesis in rats since it was first reported by Bird in 1987 [9]. Intestinal goblet cells secrete two different mucins, the sulfomucin (SUM) and the sialomucin (SIM) [10]. According to different mucin production, ACF can be classified into sulfomucin-producing ACF (SUM-ACF), sialomucin-producing ACF (SIM-ACF), and mucin-depleted foci (MDF). MDF with absent or scant secretion of mucin are positively correlated to the number of colonic tumors [11]. MDF become larger over time and spread more in the distal colon, thus they are an indicator of precancerous lesions and can be used as a short-term endpoint for CRC studies [12]. Therefore, ACF and their produced mucins can be used as good biomarkers to detect the efficacy of chemopreventive agents in colon carcinogenesis.

Djulis (*Chenopodium formosanum*) is a native cereal crop planted in Taiwan and traditionally called “ruby of cereals” for its bright red color. Djulis has many health benefits, such as anti-adipogenesis [13] and recovering liver injury [14,15]. Some studies found that the color of djulis was mainly from betalains, including betanin, isobetanin, amaranthine, and isoamaranthine, which were directly related to its antioxidant capacity [16]. Moreover, djulis contains high levels of dietary fiber, proteins, and grain-limited essential amino acids (e.g., lysine) [17].

Certain dietary fiber, as a good prebiotics, can promote the growth of the intestinal bacterial population towards a relative increase in *Bifidobacterium* and/or *Lactobacillus* species [18]. Some studies have indicated that modulation of the gut microbiota positively affects the interaction between microbiota and the host immune system, and thus may be beneficial in suppressing CRC development [19]. Moreover, emerging studies suggest that synbiotics, the combination of prebiotics and probiotics, are more effective in preventing CRC than either prebiotics or probiotics alone [20].

Our previous study demonstrated that dietary djulis inhibited the development of precancerous lesions of CRC in a carcinogen-induced animal model [21]. Besides, we also found that *Lactobacillus acidophilus* reduced inflammation in lipopolysaccharide (LPS)- and tumor necrosis factor alpha (TNF-α)-induced inflammatory colon cancer cells [22]. However, the effect of combination of djulis and probiotics and the detailed mechanism of action remain to be elucidated. The aim of this study was to investigate the preventive effect of djulis combined with *L. acidophilus* on colon carcinogenesis in a rat model.

## 2. Materials and Methods

### 2.1. Materials

Djulis was acquired from Sinfong Agritech Co. (Taipei, Taiwan). *L. acidophilus* LA-5^®^ powder was from Chr. Hansen (Hørsholm, Denmark). BD Difco^TM^ Lactobacilli MRS Broth was purchased from BD^TM^ (Franklin Lakes, NJ, USA). Methylene blue, acetic acid, and iron (III) chloride hexahydrate were purchased from Nacalai Tesque Inc. (Tokyo, Japan), Showa Chemicals Co. (Tokyo, Japan) and Shimakyu Pure Chemicals (Osaka, Japan), respectively. Caspase-3 antibody (GTX110543) was purchased from Genetex Inc. (Irvine, CA, USA). Bax antibody (Catalogue number: 2772) and Bcl-2 antibody (Catalogue number: 2870) were purchased from Cell Signaling Technology Inc. (Danvers, MA, USA). Proliferating cell nuclear antigen (PCNA) antibody (Catalogue number: 13110), Goat anti-rabbit IgG secondary antibody, peroxidase AffiniPure goat anti-mouse IgG, and COX-2 antibody (Catalogue number: ab6665) were purchased from Abcam (Cambridge, UK), Southern Biotechnology Associates, Inc. (Birmingham, AL, USA) and Jackson ImmunoResearch Inc. (West Grove, PA, USA), respectively. Antibody dilutions for caspase-3, Bax, Bcl-2, PNCA, and COX-2 were 1: 2000, 1:1000, 1:1000, 1:1000, and 1:5000, respectively. β-Actin antibody, 1,2-dimethylhydrazine (DMH), dextran sulfate sodium (DSS) salt from *Leuconostoc*, N, *N*′-dimethyl-*m*-phenylenediamine, Alcian blue, N, *N*′-dimethyl-*p*-phenylenediamine, agar, and the other chemicals were all purchased from Sigma Chemical Co. (St. Louis, MO, USA).

### 2.2. Experimental Diets

**Figure 1:**
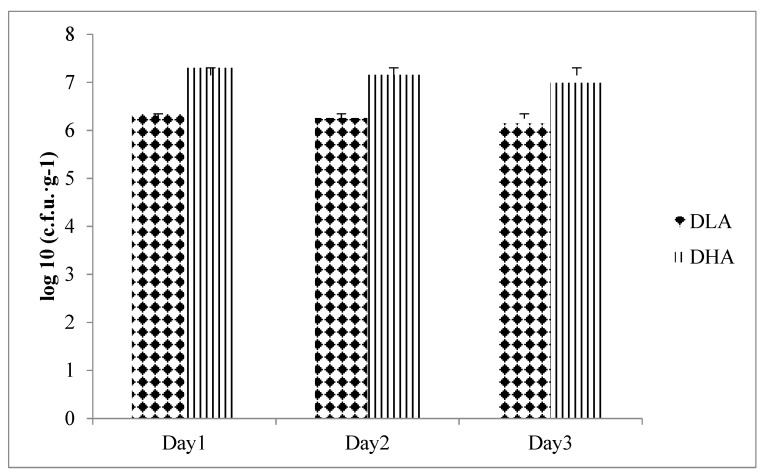
Total plate count of *Lactobacillus acidophilus* in diets. DLA, AIN-93G containing 10% djulis + 5 × 10^6^ cfu *L. acidophilus*/g; DHA, AIN-93G containing 10% djulis + 5 × 10^7^ cfu *L. acidophilus*/g.

The experimental diet was an AIN-93G-based diet containing djulis alone or in combination with *L. acidophilus*. The content of *L. acidophilus* LA-5^®^ in powder was 6 × 10^10^ colony-forming unit (cfu)/g. Diets were stored at room temperature and then analyzed for the bacteria count [23] to ensure that the doses of groups DLA and DHA were 5 × 10^6^ cfu *L. acidophilus* LA-5^®^/g and 5 × 10^7^ cfu *L. acidophilus* LA-5^®^/g, respectively (Figure 1).

### 2.3. Animals and Treatments

**Figure 2:**
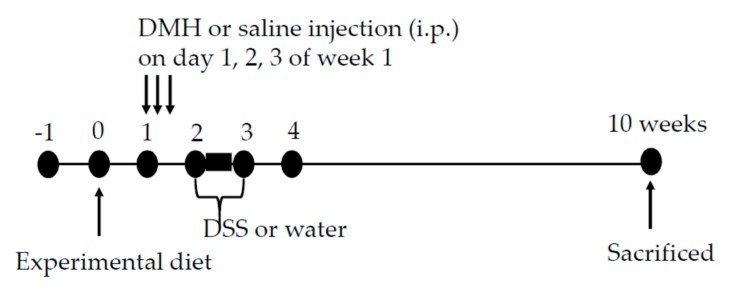
The experimental process.

The protocol used herein was approved by the Institutional Animal Care and Use Committee (IACUC) of Taipei Medical University. The approval number is LAC-2015-0400. Sixty male F344 rats (3–5 weeks old) were from National Laboratory Animal Center (Taipei, Taiwan) and housed under standard conditions (21 ± 2 °C, 40–60% humidity under a 12-h light-dark cycle). The rats had free access to diet and water. After an adaptation period (1 week), the rats were randomized into 5 groups (12 rats per group) and fed B (AIN-93G, blank), C (AIN-93G, control), D (10% djulis), DLA (10% djulis plus 5 × 10^6^ cfu/g of *L. acidophilus* LA-5^®^), and DHA (10% djulis plus 5 × 10^7^ cfu/g of *L. acidophilus* LA-5^®^) diets, respectively (Table 1). The composition of djulis and experimental diet are shown in Table 2 and Table 3, respectively. After one week of experimental diet feeding, rats in groups C, D, DLA, and DHA were given intraperitoneal injections of DMH (40 mg/kg) for 3 consecutive days during the second week of dietary treatment, and after DMH injection, these groups were treated with 3% DSS in drinking water for one week (Figure 2). The fresh experimental diets were supplied every three days. Body weight and feed consumption were measured every three days during the experimental period. After 10 weeks of feeding, all rats were sacrificed and the cecum, colons, and feces were collected and examined for precancerous lesions and biomarkers.

### 2.4. Fecal L. acidophilus Counting

Serial dilutions of feces were conducted on Lactobacilli MRS Broth-agar. The count of fecal *L. acidophilus* was determined on the above-mentioned medium [24].

### 2.5. Measurement of the Cecum

The cecum was excised, weighed, and then split open [25]. The weights of the cecum wall and content were also recorded.

### 2.6. Analysis of Colonic ACF

ACF were analyzed by the method described in our previous study [26]. The colon was removed, opened longitudinally and rinsed in saline, and then fixed flat between filter papers in 10% buffered formalin for 24 h. After being stained with 0.2% methylene blue solution for 5 min, fixed sections were placed on microscopic slides and the mucosal side was examined under a light microscope (Nikon Corp., Tokyo, Japan) at 40× magnification. Total numbers of ACF and aberrant crypts (ACs) in each focus were counted and the colonic area was calculated by NIS-Elements microscope imaging software (Nikon Corp., Tokyo, Japan). All data of ACF and AC were presented as number/cm^2^.

### 2.7. Determination of Mucin Production in ACF

The distal colons were immersed in 75% ethanol for fading after being stained with methylene blue, and then stained with high-iron diamine Alcian blue (HIDAB). The colons were observed under a light microscope (Nikon Corp., Tokyo, Japan) at 40× magnification. ACF stained bright or dark blue indicated SIM production while those stained dark brown indicated SUM production. The definition of SUM-ACF were samples with more than 85% SUM-producing cells, and for SIM-ACF were those with more than 85% SIM-producing cells. Those with not more than 85% SUM-producing or 85% SIM-producing cells were defined as mixed-type (MIX)-ACF. Furthermore, foci with very little or no secretion of any mucin were defined as MDF [27]. The colonic area was calculated by NIS-Elements microscope imaging software (Nikon Corp., Tokyo, Japan). Data of SIM-, SUM-, MIX-ACF, and MDF were presented as number/cm^2^.

### 2.8. Analysis of Protein Expression

Colon samples were homogenized in in five volumes of modified radioimmunoprecipitation assay (RIPA) buffer (0.5 M Tris-HCl, 2.5% deoxycholic acid, 1.5 M NaCl, 10% NP-40, 10 mM ethylenediaminetetraacetic acid, pH 7.4) and 10% protease inhibitor cocktail. The supernatants of homogenates were collected by centrifuging the homogenates at 10,000× *g* at 4 °C for 15 min. The total protein concentration of supernatants was confirmed by Bradford protein assay. Western blot was used for protein analysis. Protein samples were separated on 10% sodium dodecyl sulfate polyacrylamide gels (SDS-PAGE) and transferred to polyvinylidene difluoride membranes. After blocking with 5% bovine serum albumin (BSA) in Tris-buffered saline containing 1% Tween 20 (TBST), the membranes were incubated with the appropriate antibodies at 4 °C for 16 h. The membranes were then washed with TBST and incubated with anti-mouse or anti-rabbit horseradish peroxidase-conjugated secondary antibodies for 1 h. After washing with TBST, the immunocomplexes were visualized by chemiluminescence reagents and detected by chemiluminometer (BioSpectrum AC Imaging System, Ultra-Violet Products Ltd., Cambridge, United Kingdom).

### 2.9. Statistical Analysis

Data were presented as means ± SD. Differences among the experimental data were assessed by one-way analysis of variance (ANOVA) followed by Duncan’s multiple range test. All statistical analyses were carried out using the SAS software (SAS Institute, Cary, NC, USA). All *p* values < 0.05 were considered statistically significant.

## 3. Results

### 3.1. Animal Growth and Fecal L. acidophilus Count

**Figure 3:**
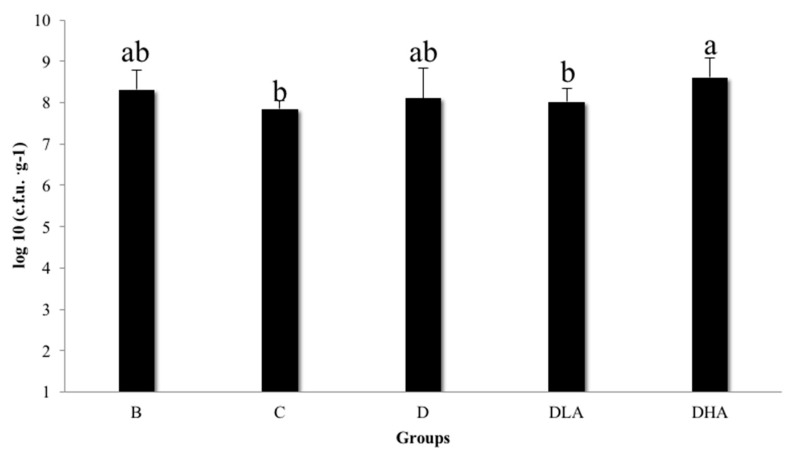
Total count of fecal *Lactobacillus acidophilus* in F344 rats. All rats except those in group B were administered with DMH/DSS. B, AIN-93G diet; C, AIN-93G diet; D, AIN-93G containing 10% djulis; DLA, AIN-93G containing 10% djulis + 5 × 10^6^ cfu *L. acidophilus*/g; and DHA, AIN-93G containing 10% djulis + 5 × 10^7^ cfu *L. acidophilus*/g. Different letters above the error bars indicate a significant difference as determined by one-way ANOVA followed by Duncan’s multiple-range test, *p* < 0.05 (*n* = 12).

There were no differences in body weight gain, feed consumption, and feed efficiency among groups (data not shown). Group C demonstrated a slight but insignificant decrease in fecal *L. acidophilus* count compared to group B (Figure 3). Group DHA had a significantly higher fecal *L. acidophilus* count than group C while the counts of fecal *L. acidophilus* were similar among groups C, D, and DLA (Figure 3).

### 3.2. Cecum Weight and pH Value

There were no differences in the cecum wall weight and relative cecum wall weight among groups (Table 4). Groups D, DLA, and DHA had a significantly higher cecum weight, relative cecum weight, cecum content weight, and relative cecum content weight than group C (Table 4). There was no difference in cecal pH value between groups B and C; however, significantly lower cecal pH values were observed in groups D, DLA, and DHA compared to group C (Supplementary Figure S1).

### 3.3. ACF in the Colon

**Figure 4:**
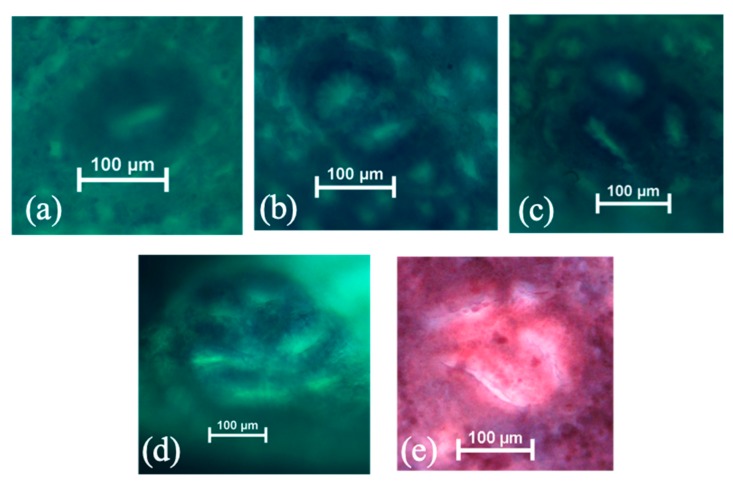
The representative images of aberrant crypt foci (ACF) with one (**a**), two (**b**), three (**c**), and six (**d**) crypts, as well as mucin-depleted foci (MDF, (**e**)) in DMH/DSS-induced male F344 rats. The original magnification is 100×.

All DMH/DSS-treated groups successfully induced ACF formation as the incidences of ACF shown in groups C, D, DLA, and DHA were all 100%. The representative images of ACF and MDF in this study are shown in Figure 4. Group DHA had a significantly lower total ACF number in the colon than group C (Table 5). According to previous studies, ACF could be distinguished into small (one to three crypts per focus) and large (four or more crypts per focus) ones [28]. Groups DLA and DHA had a significantly lower number of large ACF than group C (Table 5). ACF were mainly observed in the distal colon. Groups D, DLA, and DHA had a significantly lower number of distal ACF compared to group C (Table 6). We further examined the various sizes of ACF present in the distal colon and the results showed that groups D, DLA, and DHA had significantly lower numbers of small ACF compared to group C (Table 7). Groups D and DHA significantly inhibited the formation of large ACF in the distal colon while there was no distal large ACF in group DLA (Table 7).

### 3.4. Mucin Production by ACF and MDF

The results of HIDAB-stained colons showed that groups D, DLA, and DHA had significantly lower numbers of SIM-ACF in the distal colon compared to group C (Table 8). MDF, an advanced precancerous lesion, was induced in all DMH/DSS-treated groups (Table 9). All djulis-treated groups showed a slightly lower MDF incidence than group C while the numbers of MDF in groups D, DLA, and DHA were significantly lower compared to group C (Table 9).

### 3.5. Expression of Proliferation- and Inflammation-Related Proteins

**Figure 5:**
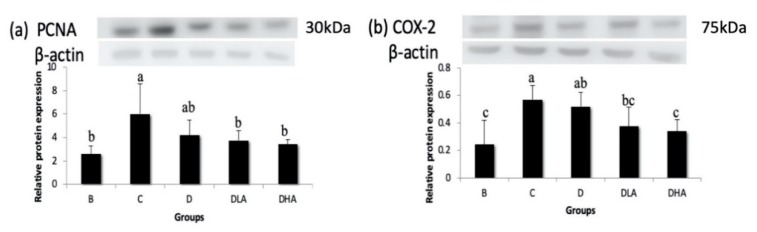
Effects of djulis and *Lactobacillus acidophilus* on the expressions of proliferating cell nuclear antigen (PCNA) (**a**) and cyclooxygenase-2 (COX-2) (**b**) in the distal colon of male F344 rats. The bars represent mean ± S.D. (*n* = 4–6). Different letters above the error bars indicate a significant difference as determined by one-way ANOVA followed by Ducan’s multiple range test, *p* < 0.05. All rats except those in group B were administered with DMH/DSS. C, AIN-93G diet; D, AIN-93G containing 10% djulis; DLA, AIN-93G containing 10% djulis + 5 × 10^6^ cfu *L. acidophilus*/g; DHA, AIN-93G containing 10% djulis + 5 × 10^7^ cfu *L. acidophilus*/g.

DMH/DSS administration (group C) significantly upregulated the expressions of PCNA (a proliferation-related protein) and COX-2 (an inflammation-related protein). Both groups DLA and DHA had significantly lower PCNA and COX-2 expressions than group C (Figure 5).

### 3.6. Expression of Apoptosis-Related Proteins

**Figure 6:**
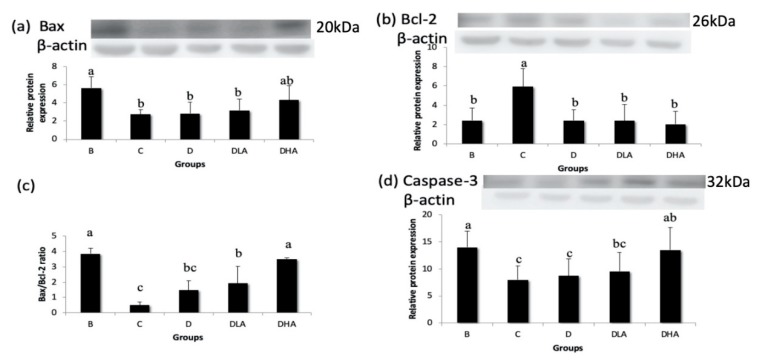
Effects of djulis and *Lactobacillus acidophilus* on the expressions of Bcl-2-associated X (Bax) (**a**), B-cell lymphoma 2 (Bcl-2) (**b**), Bax/Bcl-2 ratio (**c**), and caspase-3 (**d**) in the distal colon of male F344 rats. The bars represent mean ± S.D. (*n* = 4–6). Different letters above the error bars indicate significant difference as determined by one-way ANOVA followed by Ducan’s multiple range test, *p* < 0.05. All rats except those in group B were administered with DMH/DSS. C, AIN-93G diet; D, AIN-93G containing 10% djulis; DLA, AIN-93G containing 10% djulis + 5 × 10^6^ cfu *L. acidophilus*/g; DHA, AIN-93G containing 10% djulis + 5 × 10^7^ cfu *L. acidophilus*/g.

DMH/DSS treatment (group C) significantly downregulated pro-apoptotic Bax expression, upregulated anti-apoptotic Bcl-2 expression, and thus decreased the Bax/Bcl-2 ratio compared with group B (Figure 6). Group DHA recovered Bax expression to a level similar to group B while groups D, DLA, and DHA had significantly lower Bcl-2 expression compared with group C (Figure 6). The Bax/Bcl-2 ratio was elevated significantly in groups DLA and DHA compared with group C (Figure 6). DMH/DSS treatment (group C) also significantly downregulated the expression of pro-apoptotic caspase-3, which recovered in group DHA compared with group C (Figure 6).

## 4. Discussion

Overall, the present study showed that dietary treatment with either djulis alone or in combination with *L. acidophilus* showed a preventive effect on DMH/DSS-induced colon carcinogenesis in rats. The preventive effects could be proven by the reduction of distal ACF, the low number of MDF, and the decreased expression of Bcl-2 in all djulis-treated groups. Moreover, the synbiotic combination of djulis and *L. acidophilus* showed better inhibitory effects on the formation of large ACF and the expressions of PCNA and COX-2, as well as a better enhancing effect on the Bax/Bcl-2 ratio and caspase-3 expression compared to djulis alone.

The model of DMH/DSS-induced inflammation-associated colon carcinogenesis in rats has been widely used in many studies. Animals are often given DSS shortly after injection of DMH, which causes a decrease in the secretion of SUM-type mucin in the mucosa, impaired intestinal barrier function, induction of intestinal inflammation, and acceleration of CRC development [29]. In addition, as a promoter of CRC, DSS can significantly promote the formation of MDF [30]. Therefore, we used DMH and DSS to induce colitis-associated carcinogenesis in this study. The ACF incidence rates of all DMH/DSS-treated groups were 100%, representing successfully induced colon carcinogenesis by the DMH/DSS treatment.

ACF is a precancerous lesion of CRC in rodents and humans, which may progress to early and advanced adenomas [31,32]. As the total number of colonic ACF increased, the risk of CRC also increased [33]. In the present study, group DHA, which combined djulis with a high dose of *L. acidophilus*, significantly reduced the total ACF number compared to group C. Besides, the number of ACs in ACF can also be a marker to evaluate the progression of CRC. A previous study indicated that the greater the number of large ACF, the greater the risk of tumorigenesis [34]. In the present study, group C had the highest number of large ACF among groups, whereas the combined treatment of djulis and *L. acidophilus* in groups DLA and DHA significantly reduced the numbers of large ACF. These results suggest the potential of djulis and *L. acidophilus* in chemoprevention of CRC.

Several studies indicated that chemical carcinogens induced colon cancer progression primarily by causing damage to the colonic mucosa [35]. ACF mainly appear in the distal colon at an early stage and expand to the proximal colon during advanced progression, and thus there is a relatively high number of ACF in the middle and distal colons [36,37]. A similar result was found in this study. ACF mainly distributed in the distal colon of all DMH/DSS-treated groups. Furthermore, both djulis alone and the combination of djulis with *L. acidophilus* significantly decreased the ACF number in the distal colon. These findings demonstrate that djulis and *L. acidophilus* have the ability to prevent colon carcinogenesis through inhibiting ACF development at an early stage.

Secretion of colonic mucins may change during the progression of CRC. A previous study indicated that secretion of mucin in ACF would gradually change from SUM to SIM [38]. It was also mentioned in another study that SIM-ACF had a higher hyperplastic index in the crypt, more dysplasia, and larger abnormal crypts compared to SUM-ACF [27]. In the present study, combined treatment of djulis and *L. acidophilus* (groups D, DLA, and DHA) significantly reduced the number of SIM-ACF compared to group C. It suggests that either djulis alone or the combination of djulis with *L. acidophilus* could inhibit the progress of CRC by regulating the colonic secretion of mucins.

A previous study found that both azoxymethane (AOM) and DMH promoted the formation of MDF in the colon [39]. In the present study, groups D, DLA, and DHA had a significantly lower MDF number than group C. Moreover, group DHA had the lowest MDF incidence. MDF that exhibit absent or scant production of mucin carry alterations in cell signaling pathways and gene mutations, with a frequency similar to that observed in tumors [40]. It has been proven that the number of MDF was positively correlated to the incidence of CRC [41]. These results show that either djulis alone or the combination of djulis with *L. acidophilus* have an inhibitory effect on the progression from primary to advanced precancerous lesions during colon carcinogenesis.

The present study showed that the fecal count of *L. acidophilus* in group DHA was significantly higher than that in group C. Previous studies have shown that the induction by DMH and DSS will lead to an increase in the intestinal pH, and thus reduce the number of intestinal probiotics, such as *Lactobacillus* and *Bifidobacterium* [42]. A similar phenomenon was found in patients with irritable bowel syndrome. After treatment with *L. acidophilus* (10^9^ cfu/day) and dietary fiber (consisting of 90% inulin and 10% oligofructose) for four weeks, the content of *L. acidophilus* in the feces was significantly increased compared to the placebo group, which confirmed that synbiotics can modulate the intestinal microflora [43]. A preliminary study in our laboratory demonstrated that the combination of probiotic *L. acidophilus* and germinated brown rice rich in dietary fiber suppressed DMH/DSS-induced colonic precancerous lesions via regulation of antioxidation and apoptosis in rats [44]. These findings suggest that such a synbiotic combination may be a potential functional food or chemopreventive agent for the control of CRC [44]. Meanwhile, the present study proved that the combination of djulis and a high dose of *L. acidophilus* improved DMH/DSS-induced reduction of *L. acidophilus* in feces. Overall, it is speculated that djulis could provide prebiotic dietary fiber for promoting the growth of *L. acidophilus* in the intestine.

The cecum is the main fermentation organ in rodents. Short-chain fatty acids (SCFAs) produced by fermentation can maintain intestinal epithelial cells, reduce intestinal pH, and stimulate the growth of probiotics [45]. Studies showed that simultaneous administration of inulin and probiotics to mice increased the cecal weight, which represented a higher microbial content [46]. Femia et al. [47] pointed out that in an AOM-induced CRC rat model, the combination of inulin and probiotics (*Lactobacillus rhamnosus* and *Bifidobacterium lactis*) increased the concentration of SCFAs in the gut and inhibited the formation of ACF. Moreover, the synbiotic combination showed a stronger inhibitory effect on ACF than the probiotics or prebiotics alone [47]. In the present study, the relative cecum content weight was negatively correlated to the total ACF number in the distal colon (*r* = −0.51, *p* < 0.05) and positively correlated to caspase-3 protein expression (*r* = 0.79, *p* < 0.0001). It suggests that synbiotics may inhibit the formation of colonic precancerous lesions by promoting fermentation in the cecum.

Chronic inflammation plays an important role in cancer development in several tissues, including the large intestine. The progression of CRC is accelerated by COX-2, which may promote cell proliferation, anti-apoptosis, invasion, metastasis, angiogenesis, and drug resistance in colon cancer [48]. In the present study, the expression of inflammation-related COX-2 was increased by DMH/DSS treatment; conversely, COX-2 expression was reduced by either djulis alone or the combination of djulis with *L. acidophilus*. Moreover, the combination of djulis with a high dose of *L. acidophilus* showed the best inhibitory effect on COX-2 expression. These findings suggest that djulis and *L. acidophilus* may suppress colon carcinogenesis via an inflammation-associated pathway.

Although djulis alone had no effect on proliferation-related PCNA, the combination of djulis with *L. acidophilus* reduced PCNA expression in the present study. Besides, the number of SIM-ACF was positively correlated to PCNA expression (*r* = 0.55, *p* < 0.05). A previous study found that both inhibition of PCNA expression and promotion of caspase-3 expression were confirmed in the activation of the extrinsic pathway of apoptosis [49]. Increasing the Bax/Bcl-2 ratio or inhibiting Bcl-2 expression, both markers of apoptosis, by treatment with bioactive compounds in colon cancer cells limited their growth [50,51]. The present study found that the combination of djulis with *L. acidophilus* could increase the Bax/Bcl-2 ratio and caspase-3 expression while treatment with djulis alone had no such effect. It suggests that the combination of djulis with *L. acidophilus* may inhibit cell proliferation and promote apoptosis in the DMH/DSS-induced CRC animal model.

In this study, 10% djulis in the experimental diet is equal to 44 g djulis for a 60 kg human per day. Besides, the doses of probiotics used in the DLA and DHA groups are equal to 2.5 × 10^9^ and 2.5 × 10^10^ cfu *L. acidophilus* for a 60 kg human per day, respectively. It is easy to have these doses of probiotics from commercial yogurts, which usually contain 10^8^ to 10^10^ cfu probiotics in each bottle. Therefore, the doses of djulis and *L. acidophilus* used in this study are feasible for humans to take in daily life.

## 5. Conclusions

Djulis, a cereal grain rich in prebiotic dietary fiber and chemopreventive substances, was proven to have an anti-cancer potential in inflammation-associated colon carcinogenesis in the present study. The combination of djulis and *L. acidophilus* as synbiotics may reduce the formation of colonic precancerous lesions by regulating colon cancer biomarkers, especially by inhibiting cell proliferation and inflammation, as well as promoting apoptosis. The synbiotic combination shows the best potential for chemoprevention of colon cancer.

## Figures and Tables

**Table 1:** Experimental groups.

Group	Treatment	Diet
Blank (B)	Saline/Sterile water	AIN-93G
Control (C)	DMH/DSS	AIN-93G
Djulis (D)	DMH/DSS	AIN-93G containing 10% djulis
Djulis + low dose of *L. acidophilus* (DLA)	DMH/DSS	AIN-93G containing 10% djulis +5 × 10^6^ cfu/g of *L. acidophilus* LA-5^®^
Djulis + high dose of *L. acidophilus* (DHA)	DMH/DSS	AIN-93G containing 10% djulis +5 × 10^7^ cfu/g of *L. acidophilus* LA-5^®^

**Table 2:** The composition of djulis.

Composition	Content (g/100 g)
Moisture	10.34
Ash	6.98
Crude fat	6.43
Crude protein	11.94
Total dietary fiber	18.54
Nitrogen-free extract ^1^	45.77

^1^ Nitrogen-free extract: 100—moistu—a—crude f—crude prote—total dietary fiber.

**Table 3:** The composition of the experimental diet.

Composition	Content (g/kg Diet)				
	B	C	D	DLA	DHA
Djulis	—	—	100	100	100
*L. acidophilus* powder	—	—	—	0.083	0.83
Cornstarch	397.5	397.5	334.41	334.41	334.41
Casein	200	200	188.06	188.06	188.06
Dextrinized cornstarch	132	132	132	132	132
Sucrose	100	100	100	100	100
Soybean oil	70	70	63.57	63.57	63.57
*α*-Cellulose	50	50	31.46	31.46	31.46
Mineral mix	35	35	35	35	35
Vitamin mix	10	10	10	10	10
L-cystine	3	3	3	3	3
Choline bitartrate	2.5	2.5	2.5	2.5	2.5
*tert*-Butylhydroquinone (mg)	14	14	14	14	14

**Table 4:** Effects of djulis and *Lactobacillus acidophilus* on the weights of the cecum, cecum wall, and cecum content of male F344 rats ^1,2^.

Group ^3^	Cecum Weight (g)	Relative Cecum Weight (g/100 g BW)	Cecum Wall Weight (g)	Relative Cecum Wall Weight (g/100 g BW)	Cecum Content Weight (g)	Relative Cecum Content Weight (g/100 g BW)
B	1.6 ± 0.7 ^b^	0.4 ± 0.2 ^b^	0.5 ± 0.2 ^a^	0.14 ± 0. 05 ^a^	1.1 ± 0.6 ^b^	0.3 ± 0.2 ^b^
C	1.8 ± 0.4 ^b^	0.5 ± 0.1 ^b^	0.6 ± 0.1 ^a^	0.15 ± 0. 02 ^a^	1.2 ± 0.4 ^b^	0.3 ± 0.1 ^b^
D	2.3 ± 0.7 ^a^	0.6 ± 0.2 ^a^	0.6 ± 0.1 ^a^	0.15 ± 0. 02 ^a^	1.7 ± 0.7 ^a^	0.5 ± 0.2 ^a^
DLA	2.5 ± 0.6 ^a^	0.7 ± 0.1 ^a^	0.6 ± 0. 0 ^a^	0.16 ± 0. 01 ^a^	1.9 ± 0.6 ^a^	0.5 ± 0.1 ^a^
DHA	2.4 ± 0.5 ^a^	0.7 ± 0.1 ^a^	0.6 ± 0.1 ^a^	0.16 ± 0. 02 ^a^	1.8 ± 0.5 ^a^	0.5 ± 0.1 ^a^

^1^ All values are mean ± SD (*n* = 12). ^2^ Values with the different superscript letters in a column are significantly different from one another as determined by one-way ANOVA followed by Duncan’s multiple range test, *p* < 0.05. ^3^ All rats except those in group B were administered with DMH/DSS. B, AIN-93G diet; C, AIN-93G diet; D, AIN-93G containing 10% djulis; DLA, AIN-93G containing 10% djulis + 5 × 10^6^ cfu *L. acidophilus*/g; DHA, AIN-93G containing 10% djulis + 5 × 10^7^ cfu *L. acidophilus*/g.

**Table 5:** Effects of djulis and *Lactobacillus acidophilus* on DMH/DSS-induced ACF in the colon of male F344 rats ^1,2^.

Group ^3^	ACF Incidence (% of Animals with ACF)	Number of ACF (Number/cm^2^)	Small ACF (≤3 Crypts)	Large ACF (≥4 Crypts)
C	100%	6.5 ± 2.4 ^a^	6.1 ± 2.0 ^a^	0.4 ± 0.4 ^a^
D	100%	5.9 ± 1.9 ^ab^	5.6 ± 1.9 ^a^	0.3 ± 0.2 ^ab^
DLA	100%	5.5 ± 1.9 ^ab^	5.4 ± 1.8 ^a^	0.1 ± 0.1 ^b^
DHA	100%	5.1 ± 1.6 ^b^	4.9 ± 1.5 ^a^	0.1 ± 0.2 ^b^

^1^ Values are percentage or mean ± SD (*n* = 12). ^2^ Values with the different superscript letters in a column are significantly different from one another as determined by one-way ANOVA followed by Duncan’s multiple range test, *p* < 0.05. ^3^ All rats were administered with DMH/DSS. C, AIN-93G diet; D, AIN-93G containing 10% djulis; DLA, AIN-93G containing 10% djulis + 5 × 10^6^ cfu *L. acidophilus*/g; DHA, AIN-93G containing 10% djulis + 5 × 10^7^ cfu *L. acidophilus*/g.

**Table 6:** Effects of djulis and *Lactobacillus acidophilus* on the distribution of DMH/DSS-induced ACF in the colon of male F344 rats ^1,2^.

Group ^3^	Proximal Colon	Middle Colon	Distal Colon
C	3.2 ± 1.3 ^a^	7.4 ± 2.6 ^a^	11.8 ± 5.3 ^a^
D	2.2 ± 2.2 ^a^	7.0 ± 1.8 ^a^	8.0 ± 3.4 ^b^
DLA	2.5 ± 2.8 ^a^	6.5 ± 3.0 ^a^	7.6 ± 2.0 ^b^
DHA	2.6 ± 2.7 ^a^	6.4 ± 3.3 ^a^	6.3 ± 2.7 ^b^

^1^ All values are mean ± SD (*n* = 12). ^2^ Values with the different superscript letters in a column are significantly different from one another as determined by one-way ANOVA followed by Duncan’s multiple range test, *p* < 0.05. ^3^ All rats were administered with DMH/DSS. C, AIN-93G diet; D, AIN-93G containing 10% djulis; DLA, AIN-93G containing 10% djulis + 5 × 10^6^ cfu *L. acidophilus*/g; DHA, AIN-93G containing 10% djulis + 5 × 10^7^ cfu *L. acidophilus*/g.

**Table 7:** Effects of djulis and *Lactobacillus acidophilus* on ACF (number/cm^2^) according to the various sizes of crypts in the distal colon of male F344 rats ^1,2^.

Group ^4^	ACF with			Small ACF (≤3 Crypts)	Large ACF (≥4 Crypts)
	1 Crypt	2 Crypts	3 Crypts		
C	6.6 ± 2.3 ^a^	3.0 ± 2.3 ^a^	1.1 ± 1.3 ^a^	10.6 ± 4.3 ^a^	1.2 ± 1.1 ^a^
D	3.7 ± 2.4 ^b^	2.4 ± 1.3 ^a^	1.2 ± 0.8 ^a^	7.4 ± 3.2 ^b^	0.6 ± 0.4 ^b^
DLA	4.3 ± 1.6 ^b^	2.5 ± 0.7 ^a^	0.9 ± 0.7 ^a^	7.6 ± 2.0 ^b^	— ^3^
DHA	3.1 ± 1.6 ^b^	2.0 ± 1.1 ^a^	0.8 ± 1.1 ^a^	6.0 ± 2.6 ^b^	0.4 ± 0.5 ^b^

^1^ All values are mean ± SD (*n* = 12). ^2^ Values with the different superscript letters in a column are significantly different from one another as determined by one-way ANOVA followed by Duncan’s multiple range test, *p* < 0.05. ^3^ No ACF observed. ^4^ All rats were administered with DMH/DSS. C, AIN-93G diet; D, AIN-93G containing 10% djulis; DLA, AIN-93G containing 10% djulis + 5 × 10^6^ cfu *L. acidophilus*/g; DHA, AIN-93G containing 10% djulis + 5 × 10^7^ cfu *L. acidophilus*/g.

**Table 8:** Effects of djulis and *Lactobacillus acidophilus* on DMH/DSS-induced ACF according to the type of mucin produced by foci in the distal colon of male F344 rats ^1,2^.

Group ^3^	Number of ACF Producing ^4^ (number/cm^2^)		
	SUM ^5^	MIX	SIM
C	0.2 ± 0.4 ^a^	0.2 ± 0.5 ^a^	10.5 ± 4.2 ^a^
D	0.1 ± 0.2 ^a^	0.1 ± 0.2 ^a^	7.5 ± 3.4 ^b^
DLA	0.4 ± 0.6 ^a^	0.0 ± 0.0 ^a^	7.3 ± 2.1 ^b^
DHA	0.4 ± 0.6 ^a^	0.1 ± 0.2 ^a^	5.8 ± 2.5 ^b^

^1^ All values are mean ± SD (*n* = 12). ^2^ Values with the different superscript letters in a column are significantly different from one another as determined by one-way ANOVA followed by Duncan’s multiple range test, *p* < 0.05. ^3^ All rats were administered with DMH/DSS. C, AIN-93G diet; D, AIN-93G containing 10% djulis; DLA, AIN-93G containing 10% djulis + 5 × 10^6^ *L. acidophilus*; DHA, AIN-93G containing 10% djulis + 5 × 10^7^ *L. acidophilus*. ^4^ SUM, sulfomucin; MIX, mixed sulfumucin and sialomucin; SIM, sialomucin. ^5^ Number of rats with SUM-ACF, MIX-ACF, or SIM-ACF.

**Table 9:** Effects of djulis and *Lactobacillus acidophilus* on DMH/DSS-induced MDF in the colon of male F344 rats ^1,2^.

Group ^3^	MDF Incidence (Number of Rats with MDF/Total Rats)	Number of MDF (Number/cm^2^)	MDF Multiplicity (Number of AC/MDF)
C	67% (8/12)	0.42 ± 0.38 ^a^	1.4 ± 0.5 ^a^
D	42% (5/12)	0.10 ± 0.14 ^b^	1.2 ± 0.4 ^a^
DLA	33% (4/12)	0.09 ± 0.14 ^b^	1.0 ± 0.0 ^a^
DHA	8% (1/12)	0.01 ± 0.04 ^b^	1.0 ± 0.0 ^a^

^1^ All values are mean ± SD (*n* = 12). ^2^ Values with the different superscript letters in a column are significantly different from one another as determined by one-way ANOVA followed by Duncan’s multiple range test, *p* < 0.05. ^3^ All rats were administered with DMH/DSS. C, AIN-93G diet; D, AIN-93G containing 10% djulis; DLA, AIN-93G containing 10% djulis + 5 × 10^6^ cfu *L. acidophilus*/g; DHA, AIN-93G containing 10% djulis + 5 × 10^7^ cfu *L. acidophilus*/g.

## Supplementary Materials

The following are available online at https://www.mdpi.com/2072-6643/12/1/103/s1, Figure S1: Cecal pH value of F344 rats.
