# Supplementary material for: Synbiotic Combination of Djulis (Chenopodium formosanum) and Lactobacillus acidophilus Inhibits Colon Carcinogenesis in Rats
Source: Nutrients. 2019 Dec 30;12(1):103. doi: 10.3390/nu12010103 (PMC7019357; doi:10.3390/nu12010103)
Supplement: Supplementary file 1 [file nutrients-12-00103-s001.pdf]

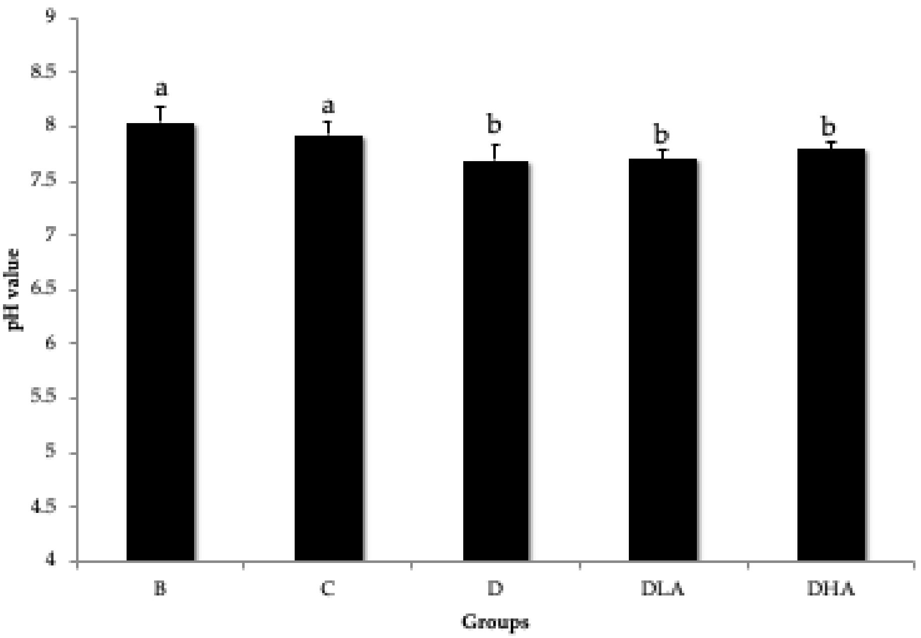

Supplementary Figure 1. Cecal pH value of F344 rats. All rats except those in group B were administered with DMH/DSS. B, AIN-93G diet; C, AIN-93G diet; D, AIN-93G containing 10% djulis; DLA, AIN-93G containing 10% djulis + 5 $\times 10^6$ cfu *L. acidophilus*/g; and DHA, AIN-93G containing 10% djulis + 5 $\times 10^7$ cfu *L. acidophilus*/g. Different letters above the error bars indicate a significant difference as determined by one-way ANOVA followed by Duncan's multiple-range test, $p < 0.05$ ($n = 12$).
